# Association of *EGLN2 rs10680577* Polymorphism with the Risk and Clinicopathological Features of Patients with Prostate Cancer

**DOI:** 10.31557/APJCP.2020.21.5.1221

**Published:** 2020-05

**Authors:** Nahid Rahimi, Mahsa Azizi, Gholamreza Bahari, Behzad Narouie, Mohammad Hashemi

**Affiliations:** 1 *Department of Clinical Biochemistry, School of Medicine, Zahedan University of Medical Sciences, Zahedan, Iran. *; 2 *Children and Adolescent Health Research Center, Zahedan University of Medical Sciences, Zahedan, Iran. *; 3 *Urology and Nephrology Research Center, Department of Urology, Shahid Labbafinejad Medical Center, Shahid Beheshti University of Medical Sciences, Tehran, Iran. *; 4 *Genetics of Non-communicable Disease Research Center, Zahedan University of Medical Sciences, Zahedan, Iran.*

**Keywords:** EGLN2- RERT, lncRNA, prostate cancer, polymorphism, indel

## Abstract

Several studies have evaluated the association between *EGLN2 4-bp* insertion/deletion (ins/del) polymorphism (*rs10680577*) and many cancers. However, up to date, no study has inspected the impact of *rs10680577* polymorphism on prostate cancer (PCa) risk. This case-control study was achieved on 170 pathologically confirmed PCa patients and 196 cancer free men to inspect whether *rs10680577* variant is related to the risk and clinicopathological features of patients with PCa. Genotyping was performed by mismatched polymerase chain reaction-restriction fragment length polymorphism (PCR-RFLP). The findings did not support an association between the variant with the risk and clinicopathological characteristics of PCa patients. When we pooled our results with six preceding studies, the findings suggested that *rs10680577* variant significantly augmented the risk of overall cancer in heterozygous (OR=1.38, 95 % CI=1.26-1.52, p<0.00001, ins/del vs ins/ins), homozygous (OR=1.66, 95 % CI=1.05-2.61, p=0.029, del/del vs ins/ins), codominant (OR=1.44, 95%CI=1.32-1.58, p<0.00001, ins/del+del/del vs ins/ins), and allele (OR=1.32, 95%CI=1.18-1.49, p<0.00001, del vs ins) genetic models. Additional well designed studies with larger sample sizes are necessary to confirm our findings.

## Introduction

Prostate cancer (PCa) is one of the most common cancer among men globally (Jemal et al., 2011). The precise mechanisms underlying PCa development is largely unknown. Mounting evidence suggests that genomic and environmental factors play a role in development and progression of PCa (Cunningham et al., 2003; Chokkalingam et al., 2007; Zhou et al., 2015; Sattarifard et al., 2018). Small insertions/deletions (indels), the second most common form of genetic variations in human genome, have been linked to cancer development (Mullaney et al., 2010; Hashemi et al., 2018a; Hashemi et al., 2018c; Hashemi et al., 2018d). 

*EGLN2 (Egl nine homolog 2)* gene which is located on chromosome 19 (19q13.2) encodes prolyl hydroxylases 1 (PHD1) (Ryan et al., 2014). 

Hypoxia, a main characteristic of solid tumors, leads to alterations of gene expression in tumor cells to adapt to the hypoxic environment (Brahimi-Horn et al., 2007). The hypoxia-inducible factor 1 (HIF-1), a key transcriptional activator is induced by hypoxia (Semenza, 1999). The HIF-1 plays a critical role in the development of solid tumors and in coordinating the cellular response to hypoxia and oxygen homeostasis (Maxwell and Ratcliffe, 2002; Semenza, 2007; Kaelin and Ratcliffe, 2008). The level of HIF-1 is tightly regulated by three PHDs (PHD1, PHD2 and PHD3) (Appelhoff et al., 2004; Willam et al., 2004). In normoxia condition HIF is hydroxylated at specific residues by PHDs which uses oxygen as a substrate. Hydroxylated HIF binds to a protein called Von Hippel Lindau protein (VHL) for its degradation, while in hypoxic situation, stabilization and nuclear translocation occur, leading to oncogenes activation (Appelhoff et al., 2004; Stolze et al., 2006; Pezzuto and Carico, 2018). 

Several studies investigated the correlation between EGLN2 4-bp ins/del polymorphism (*rs10680577*) and susceptibility to various cancer comprising breast cancer (Hashemi et al., 2018b), colorectal cancer (Li et al., 2017), gastric cancer (Wang et al., 2014), hepatocellular carcinoma (HCC) (Zhu et al., 2012), and lung cancer (Che et al., 2014; Zhu et al., 2018), As far as we know, there is no data concerning the impact of EGLN2 4-bp ins/del polymorphism on PCa susceptibility. Consequently, the current study aimed to assess the impact of this variant on PCa development. 

## Materials and Methods

This case-control study conducted on 170 histologically confirmed PCa patients and 196 cancer free men. The study design and enrollment procedure have been explained previously (Hashemi et al., 2017a; Hashemi et al., 2017b; Sattarifard et al., 2018). The study was approved by the Zahedan University of Medical Sciences ethics committee and all participants were asked to provide their written informed consent. Whole blood samples were collected in EDTA tube, and genomic DNA was purified by salting out method.

*Genotyping*

Genotyping of EGLN2 4-bp ins/del (*rs10680577*) polymorphism was done by mismatch polymerase chain reaction-restriction fragment length polymorphism (PCR-RFLP) as described previously (Hashemi et al., 2018b). The forward and reverse primers were 5`-CCGTTATAAAAGATACTTATGTAAATCAC-3` and 5`-TTGGAATCAAGTGGCGTCG-3`, respectively. PCR was achieved using Prime Taq Premix (Genet Bio, Korea) and the PCR products were digested by AleI restriction enzyme. The del allele digested and created 224 and 31 bp fragments, whereas the ins allele remained undigested (259 bp).

*Statistical analysis*

All analyses were conducted with SPSS 22 statistical package. The χ^2^ and independent sample t-test were used for categorical and continuous data, respectively. Odds ratios (ORs) and 95% confidence intervals (95% CIs) was estimated by logistic regression analysis. P value < 0.05 was considered statistically significant. 

*Pooled analysis*

Pooling of our outcomes with six previous published studies was done using STATA 14.1 software. Electronic databases were searched for all articles describing the relationship between EGLN2 4-bp ins/del polymorphism and cancer susceptibility. The characteristic of study included into pooled analysis is shown in Table 3. The relationship between EGLN2 polymorphism and cancer risk was assessed by pooled ORs and their 95% CIs. The significance of the pooled OR was assessed by the Z-test, and P<0.05 was considered to be statistically significant. Heterogeneity between studies was determined by I2 test and Q test. The I2≥50% or PQ< 0.1 showed the presence of heterogeneity. If heterogeneity exists the random effect model was applied. We determined publication bias using Begg’s funnel plot and Egger’s test. Sensitivity analyses were conducted in order to assess the data stability. 

## Results

The study group consisted of 170 histologically confirmed PCa (mean age: 61.2±6.6 years) and 196 cancer free men (mean age: 64.5±8.9 years). Statistically significant difference was observed between cases and controls groups regarding age (p<0.05). The frequency distribution of genotype and allele is shown in Table 1. The results indicated that EGLN2 4-bp ins/del polymorphism was not correlated with PCa susceptibility in heterozygous (OR=0.98, 95%CI=0.61-1.57, p=0.816), homozygous (OR=0.50, 95%CI=0.21-1.21, p=0.126) dominant (OR=0.91, 95%CI= 0.58-1.45, p=0.695, recessive (OR=1.95, 95%CI=0.87-4.41, p=107) and allele (OR=0.92, 95%CI=0.69-1.25, p=0.649) genetic models. 

The relationship between the variant and clinicopathological features such as age, stage, prostate specific antigen (PSA) level, Gleason score, perineural invasion, and surgical margin were determined (Table 2). The results indicated no significant relationship between the variant and clinicopathological features.

*Main pooled analysis results*

**Figure 1:**
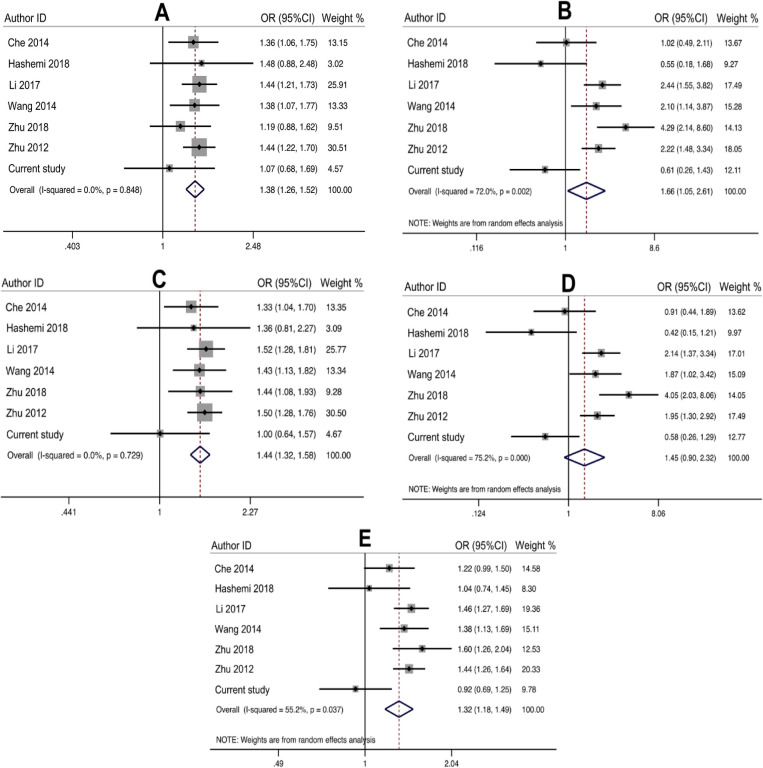
The Forest Plot for the Relationship between EGLN2 4-bp ins/del Polymorphism and Cancer Susceptibility for ins/del vs ins/ins (A), del/del vs ins/ins (B), ins/del+del/del vs ins/ins (C), dels/del vs ins/del+del/del (D), and del vs ins (E).

The pooled results with six previous published studies support an association between 4-bp ins/del polymorphism of EGLN2 and cancer susceptibility. The variant positively associated with overall cancer susceptibility in heterozygous (OR=1.38, 95 % CI=1.26-1.52, p<0.00001, ins/del vs ins/ins), homozygous (OR=1.66, 95 % CI=1.05-2.61, p=0.029, del/del vs ins/ins), codominant (OR=1.44, 95%CI=1.32-1.58, p<0.00001, ins/del+del/del vs ins/ins), and allele (OR=1.32, 95%CI=1.18-1.49, p<0.00001, del vs ins) inheritance model (Table 4 and Figure 1).

Heterogeneity between the studies comprised in the pooled analysis is indicated in Table 2. The findings suggested no heterogeneity in heterozygous and dominant genetic models. 

Begg’s funnel plot and Egger’s test noticed no publication bias in all genetic models except in dominant (Table 4).

We executed sensitivity analysis to evaluate the influence of each study on the overall estimate. The pooled ORs were not substantially changed except in homozygous model, indicating that the present pooled analysis is stable and reliable.

**Table 1:** Genotype and Allele Frequencies of *EGLN2* rs10680577 (4-bp ins/del) Polymorphism in PCa and Controls

4-bp ins/del polymorphism	Case n (%)	Control n (%	*OR (95%CI)	*P
Codominant				
ins/ins	51 (30.0)	59 (30.1)	1	-
ins/del	109 (64.1)	118 (60.2)	0.98 (0.61-1.57)	0.816
del/del	10 (5.9)	19 (9.7)	0.50 (0.21-1.21)	0.126
Dominant				
ins/ins	51 (30.0)	59 (30.1)	1	-
ins/del+del/del	119 (70.0)	137 (69.9)	0.91 (0.58-1.45)	0.695
Recessive				
Ins/del+ins/ins	160 (94.1)	177 (90.3)	1	-
De/del	10 (5.9)	19 (9.7)	1.95 (0.87-4.41)	0.107
Allele				
ins	211 (62.0)	236 (60.2)	1	-
del	129 (38.0)	156 (39.8)	0.92 (0.69-1.25)	0.649

*Adjusted by age

**Table 2:** Association between EGLN2 4-bp ins/del Polymorphism and Clinical Characteristics of Prostate Cancer Patients

Characteristic of patients	EGLN2 4-bp ins/del			p
	Ins/ins	Ins/del	Del/del	
Age at diagnosis (years, n)				0.32
≤60	22	54	7	
>60	28	55	3	
Stage				0.554
pT1	2	5	1	
pT2a	3	20	2	
pT2b	2	8	1	
pT2c	30	50	5	
pT3a	3	6	1	
pT3b	10	20	0	
PSA level at diagnosis (ng/ml), n				0.923
≤4	1	1	0	
4-10	26	54	6	
>10	23	54	4	
Gleason score, n				0.228
≤7	40	84	10	
>7	10	25	0	
Perineural invasion, n				0.567
Positive	31	72	5	
Negative	19	37	5	
Surgical margin, n				0.883
Positive	17	40	3	
Negative	33	69	7	

**Table 3:** Characteristics of All Studies Included in the Meta-Analysis

Author	Year	Country	Ethnicity	Cancer type	Source of control	Genotyping Method	Case/control	Cases					Controls				
								Ins/ins	Ins/del	del/del	ins	del	Ins/ins	Ins/del	del/del	ins	del
Che	2014	China	Asian	NSLC	HB	PCR-PAGE	406/812	241	154	11	636	176	536	252	24	1324	300
Hashemi	2018	Iran	Asian	Breast cancer	HB	PCR-RFLP	134/154	35	94	5	164	104	50	91	13	191	117
Li	2017	China	Asian	CRC	HB	PCR-PAGE	1008/1240	571	383	54	1525	491	825	383	32	2033	447
Wang	2014	China	Asian	Gastric cancer	HB	PCR-PAGE	415/830	235	159	21	629	201	541	266	23	1348	312
Zhu	2018	China	Asian	Lung cancer	HB	PCR-PAGE	376/419	222	117	37	561	191	283	125	11	691	147
Zhu	2012	China	Asian	HCC	HB	PCR-PAGE	1067/1692	607	406	54	1620	514	1125	522	45	2772	612
Current study		Iran	Asian	Prostate cancer	HB	PCR-RFLP	170/196	51	109	10	211	129	59	118	19	236	156

**Table 4:** The Pooled ORs and 95%CIs for the Association between EGLN2 4-bp ins/del Polymorphism and Cancer Susceptibility

Genetic model	Association test			Heterogeneity test			Publication bias	
	OR (95%CI)	Z	p	χ^2^	I^2^(%)	P	Egger’s test p	Begg’s test p
ins/del vs ins/ins	1.38 (1.26-1.52)	6.98	<0.00001	2.68	0	0.848	0.109	0.051
del/del vs ins/ins	1.66 (1.05-2.61)	2.18	0.029	21.46	72	0.002	0.115	0.099
ins/del+del/del vs ins/ins	1.44 (1.32-1.58)	8.15	<0.00001	3.61	0	0.729	0.044	0.099
del/del vs ins/del+ins/ins	1.45 (0.90-2.32	1.54	0.12	24.15	75	0	0.133	0.099
del vs ins	1.32 (1.18-1.49)	4.73	<0.00001	13.39	55	0.04	0.081	0.176

## Discussion

Prolyl hydroxylases 1 (PHD1) encoded by EGLN2 gene is involved in the catalyze degradation of HIF-1 by prolyl hydroxylation of specific residues. Several studies examined the role of EGLN2 4-bp ins/del polymorphism and the risk of some cancers (Zhu et al., 2012; Che et al., 2014; Wang et al., 2014; Li et al., 2017; Hashemi et al., 2018b; Zhu et al., 2018). In the current study, for the first time, we inspected the correlation between EGLN2 4-bp ins/del polymorphism with the risk and clinicopathological characteristic of PCa. Our findings revealed no association between this variant and susceptibility as well as clinicopathological features of PCa patients. Furthermore, pooled analysis of our outcomes with six previous published studies indicated a significant association between the variant and risk of overall cancer in heterozygous, homozygous, codominant, and allele genetic models.

Long non-coding RNAs (lncRNAs), a class of non-coding transcripts longer than 200 nucleotides, are involved in epigenetic, transcriptional and post-transcriptional regulation of gene expression (Ponting et al., 2009). Growing evidence revealed that dysregulation expression of lncRNA contributes to the development and progression of various cancer for their function as proto-oncogene or anti-oncogene (Pibouin et al., 2002; Calin et al., 2007; Lin et al., 2007; He et al., 2016; Tian et al., 2016; Pei et al., 2017). 

RERT-lncRNA, with 2,849 base pairs in length, is located within the proximal promoter of EGLN2, and a 4-bp ins/del polymorphism *(rs10680577*) is within PERT-lncRNA (Zhu et al., 2012). *As rs10680577* variant is positioned within the RERT-lncRNA, it is reasonable that this variant may influence the expression level of RERT-lncRNA by affecting its folding structures. Recently, Zhu et al., (2018) reported that 4-bp ins/del polymorphism (*rs10680577*) affect the expression of EGLN2 and PERT-lncRNA. They found that the ins/del+del/del genotype carriers had increased expressions level of RERT-lncRNA as well as EGLN2. 

In conclusion, our findings proposed that EGLN2 4-bp ins/del polymorphism was not correlated with susceptibility and clinicopathological features of PCa in an Iranian population. Pooled analysis of our findings with previously published studies designated that 4-bp ins/del variant significantly augmented the risk of overall cancer. 
